# Associations of sleep disturbance with ADHD: implications for treatment

**DOI:** 10.1007/s12402-014-0151-0

**Published:** 2014-08-17

**Authors:** Allan Hvolby

**Affiliations:** Department of Child and Adolescent Psychiatry, Psychiatry of Southern Denmark, Gl. Vardevej 101, 6715 Esbjerg N, Denmark

**Keywords:** Sleep, ADHD, Stimulant, Amfetamine, Methylphenidate, Atomoxetine

## Abstract

Attention-deficit/hyperactivity disorder (ADHD) is commonly associated with disordered or disturbed sleep. The relationships of ADHD with sleep problems, psychiatric comorbidities and medications are complex and multidirectional. Evidence from published studies comparing sleep in individuals with ADHD with typically developing controls is most concordant for associations of ADHD with: hypopnea/apnea and peripheral limb movements in sleep or nocturnal motricity in polysomnographic studies; increased sleep onset latency and shorter sleep time in actigraphic studies; and bedtime resistance, difficulty with morning awakenings, sleep onset difficulties, sleep-disordered breathing, night awakenings and daytime sleepiness in subjective studies. ADHD is also frequently coincident with sleep disorders (obstructive sleep apnea, peripheral limb movement disorder, restless legs syndrome and circadian-rhythm sleep disorders). Psychostimulant medications are associated with disrupted or disturbed sleep, but also ‘paradoxically’ calm some patients with ADHD for sleep by alleviating their symptoms. Long-acting formulations may have insufficient duration of action, leading to symptom rebound at bedtime. Current guidelines recommend assessment of sleep disturbance during evaluation of ADHD, and before initiation of pharmacotherapy, with healthy sleep practices the first-line option for addressing sleep problems. This review aims to provide a comprehensive overview of the relationships between ADHD and sleep, and presents a conceptual model of the modes of interaction: ADHD may cause sleep problems as an intrinsic feature of the disorder; sleep problems may cause or mimic ADHD; ADHD and sleep problems may interact, with reciprocal causation and possible involvement of comorbidity; and ADHD and sleep problems may share a common underlying neurological etiology.

## Introduction

Attention-deficit/hyperactivity disorder (ADHD) is a common neurodevelopmental disorder that has been estimated to affect approximately 5.3 % of children and adolescents worldwide (Polanczyk et al. 2007) and to persist into adulthood in approximately two-thirds of patients (Spencer et al. 1998; Wender 1998). Inattention, hyperactivity and impulsiveness are recognized as the symptoms of ADHD according to the current diagnostic criteria [*Diagnostic and Statistical Manual of Mental Disorders* (DSM)*, Fifth Edition* (American Psychiatric Association 2013; Casas et al. 2013) and *International Classification of Diseases and Related Health Problems, 10th Revision* (ICD-10) (World Health Organization 1992)] and are associated with characteristic behavioral difficulties and impairments of day-to-day functioning. Although diagnosis relies on observations made while patients are awake, the prevalence of sleep disturbances in individuals with ADHD is reported to be in the range 25–55 % (Corkum et al. 1998; Hodgkins et al. 2013; Owens 2005; Sung et al. 2008). In a recent Australian study, 62 % of children with ADHD had moderate or severe sleep problems and 22 % took sleep medications during the 1-week observation period (Efron et al. 2014). Indeed, high nocturnal activity and disordered sleep were defining characteristics of ‘hyperkinetic reaction in childhood’ or ‘attention deficit disorder’ in earlier versions of the DSM (American Psychiatric Association 1980; Barkley 1990; Sadeh et al. 2006; Spruyt and Gozal 2011).

The association of sleep with ADHD is multifaceted and complex. Problems with sleep may be an intrinsic feature of ADHD, or may both exacerbate and be exacerbated by the symptoms of the disorder. Problems with sleep can, however, also lead to the development of ADHD or ADHD-like symptoms, potentially resulting in misdiagnosis (Cortese et al. 2006b; Owens 2008). The effects of restricted, disordered or disturbed sleep can manifest as symptoms, behaviors or functional impairments that are remarkably similar to those of ADHD (Beebe 2006; Gruber 2009; O’Brien 2009). The interrelationships are further complicated by the use of psychostimulant medications to treat ADHD, which impair sleep in some patients (Spruyt and Gozal 2011) but paradoxically (Bradley 1937) improve sleep in others via a calming effect (Jerome 2001; Kinsbourne 1973; Kooij et al. 2001; Kratochvil et al. 2005). For these reasons, it has been recommended that primary sleep disorders should be ruled out before initiating ADHD medication (Cortese et al. 2013a; Lecendreux and Cortese 2007). Behavioral interventions targeted at improving sleep may benefit some patients (Cortese et al. 2013a) and should form part of the multimodal ADHD management plan recommended for patients receiving pharmacotherapy (Graham et al. 2011; Lecendreux and Cortese 2007; Wolraich et al. 2011).

Psychiatric comorbidities are common in children with ADHD: up to 87 % of children with an ADHD diagnosis have at least one comorbidity, and 20 % have three or more comorbid conditions (Hodgkins et al. 2013; Rowland et al. 2002; Spruyt and Gozal 2011). Psychiatric illnesses such as bipolar disorder, autism, post-traumatic stress disorder and obsessive compulsive disorder often occur coincidently with ADHD, and are also associated with sleep problems, which may both result from and exacerbate comorbid psychiatric symptoms (Ivanenko et al. 2004). Problems with sleep are likely to have adverse effects on health-related quality of life for children with ADHD and their families (Hvolby et al. 2008; Saxby and Morgan 1993) and may also contribute to the development of comorbid anxiety, depression or oppositional defiant disorder (Breslau et al. 1996; Hvolby et al. 2008; Mick et al. 2000). The interactions of comorbid disorders and associated medications with ADHD and sleep disturbances are therefore important to consider when managing patients.

The complex, multidirectional interactions of sleep with ADHD, medication and psychiatric comorbidities remain unclear despite extensive research. The reciprocal nature of the relationships between ADHD and sleep may reflect the functional and neuroanatomical overlap between brain regions involved in attention, arousal and sleep regulation (Owens et al. 2013; Owens 2008). This review provides a broad but comprehensive overview of the relationships between ADHD and sleep, with the aims of fostering greater understanding of the sleep-related issues faced by many individuals with ADHD and of informing the pharmacological and non-pharmacological management of the disorder. In support of these aims, this article presents a conceptual model of the potential interactions of sleep with ADHD which is intended as an aid in the interpretation of evidence related to the interactions of sleep problems with ADHD and with medications used to treat ADHD.

## Measuring sleep in patients with ADHD

Objective measures (polysomnography, actigraphy and the multiple sleep latency test [MSLT]) and subjective measures (e.g., parent- or self-rated questionnaires and diaries) are used to assess sleep in patients with ADHD.

### Polysomnography

Polysomnography involves simultaneous and continuous measurement of multiple physiological parameters, and is usually conducted in a sleep laboratory. A combination of electroencephalography, electrocardiography, electromyography, electrooculography, pneumography and pulse oximetry is typical, sometimes together with audiovisual recordings.

Polysomnography is considered the ‘gold standard’ for the objective measurement of sleep (Cortese et al. 2009; Owens 2008), but is subject to a number of limitations. Children’s sleep patterns may be affected by the unfamiliar environment of the sleep laboratory or by the recording apparatus (Beebe 2011; Bessey et al. 2013). At least some polysomnographic parameters are subject to ‘first-night effects,’ whereby sleep characteristics on a single night may differ from those recorded on subsequent nights (Katz et al. 2002; Kirov et al. 2012; Lorenzo and Barbanoj 2002; Sadeh et al. 2006; Scholle et al. 2003).

Three published meta-analyses of polysomnographic studies have investigated sleep in children with ADHD and typically developing controls (Cortese et al. 2006a, 2009; Sadeh et al. 2006). In a meta-analysis of 11 studies, the only statistically significant polysomnographic finding was a higher incidence of periodic limb movements in sleep (PLMS) in children with ADHD than in controls (Sadeh et al. 2006). A subsequent meta-analysis of studies in non-medicated patients included 5 of these 11 studies and 4 additional polysomnographic studies (Cortese et al. 2009), and updated a previous analysis by the same group (Cortese et al. 2006a). Statistically significant polysomnographic findings were lower sleep efficiency, higher apnea–hypopnea index (AHI) and a larger number of sleep stage shifts per hour in children with ADHD than in controls (Cortese et al. 2009). Data on limb movements could not be pooled in this meta-analysis, but the authors noted that the included studies were consistent in describing elevations in indices of PLMS or general sleep movements in children with ADHD compared with controls (Cortese et al. 2006a, 2009). Since publication of these meta-analyses, four individual polysomnographic studies in un-medicated children with ADHD versus controls have each reported no statistically significant differences in any polysomnographic index, including PLMS, AHI and sleep efficiency (Choi et al. 2010; Gruber et al. 2012a; Prihodova et al. 2010, 2012), and one study has reported statistically significant differences in almost all of the polysomnographic parameters assessed (Silvestri et al. 2009). This study also indicated that children with hyperactive symptoms had a higher mean PLMS index than those with predominantly inattentive ADHD (Silvestri et al. 2009). Polysomnographic data in adults with ADHD are scarce (Yoon et al. 2012), with one study reporting no significant differences (Philipsen et al. 2005) and one reporting significant differences in four indices, compared with controls (Sobanski et al. 2008).

Several studies have reported changes in rapid eye movement (REM) sleep in children and adolescents with ADHD (Golan et al. 2004; Gruber et al. 2009; Kirov et al. 2004; O’Brien et al. 2003a, b). Inter-study inconsistencies in whether REM sleep is increased or decreased in patients with ADHD compared with controls have been ascribed to changes in REM sleep during maturation (Kirov and Brand 2014). However, three meta-analyses of polysomnographic studies found no significant alterations in REM sleep parameters in children with ADHD compared with controls (Cortese et al. 2006a, 2009; Sadeh et al. 2006). The most recent and inclusive of these ruled out inter-study heterogeneity as being responsible for the lack of any detectable pooled difference in REM sleep parameters (Cortese et al. 2009).

In summary, the available evidence from polysomnographic studies is most concordant for associations of ADHD with apnea/hypopnea and PLMS/nocturnal motor activity in children (Cortese et al. 2006a; Spruyt and Gozal 2011). The number of studies is limited, and some parameters have been reported in only one or two studies, indicating a need for additional research (Cortese et al. 2009). For example, two studies of sleep microstructure (the cyclic alternating pattern) in patients with ADHD have yielded inconsistent results (Miano et al. 2006; Prihodova et al. 2012). Furthermore, current polysomnographic indices may not be sensitive enough to detect subtle patterns of sleep fragmentation (Owens et al. 2013; Yoon et al. 2012). For example, episodes of PLMS in children with ADHD are reportedly characterized by atypical, low periodicity movements which may not register on all polysomnographic indices of PLMS, yet these may be more likely than highly periodic, stereotypical PLMS to be associated with arousal and sleep disturbance (Ferri et al. 2013).

Polysomnographic studies are generally small (typically 20–30 participants per arm), and there is a need for larger-scale, multicenter studies (Cortese et al. 2009; Sadeh et al. 2006). Sleep parameters in children with ADHD are influenced by age, gender, comorbidities, ADHD diagnostic criteria and subtype, and the inclusion of an adaptation night (to compensate for first-night effects), but these variables are not controlled in many studies (Sadeh et al. 2006). In particular, the different diagnostic criteria used in different studies may be an important factor underlying the inconsistency of polysomnographic findings across studies (Gomes et al. 2013; Kirov and Brand 2014; Kirov et al. 2004). Furthermore, children with ADHD often show marked intra-individual variability and instability in sleep parameters, rather than a consistent level of impairment (Gruber and Sadeh 2004; Gruber et al. 2000; Hvolby et al. 2008; Lecendreux and Cortese 2007; Lecendreux et al. 2000; Moreau et al. 2013; Prihodova et al. 2010).

### Actigraphy

Actigraphy involves wearing a sensor, usually on the wrist, to measure motor activity. Current devices are small, lightweight, unobtrusive and convenient. Despite the increasing use of actigraphy in pediatric studies, variations remain in the device used, the point of attachment, the parameters measured and the method for storing and analyzing the signals (Meltzer et al. 2012). Actigraphy is unable to provide information on sleep architecture, PLMS, snoring or apnea/hypopnea. Compared with polysomnography, actigraphy may overestimate waking after sleep onset and underestimate total sleep time, and may also underestimate sleep onset latency (presumably because immobility generally precedes sleep) (Spruyt et al. 2011). Nevertheless, actigraphy has the unique advantage of providing a non-invasive means of measuring sleep–wake patterns objectively over extended periods of time under everyday conditions.

**Fig. 1:**
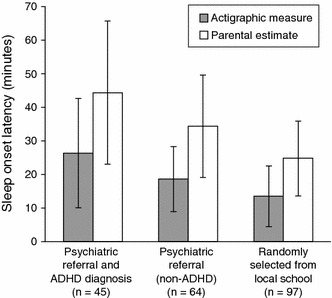
Sleep onset latency assessed by parental estimation and actigraphy (Hvolby et al. 2008). Data are shown as means ± standard deviations. Differences between the three groups were statistically significant for both the actigraphic measure (*p* < 0.01) and the parental measure (*p* < 0.001), as was the difference between the two measures (*p* < 0.001) across all groups (three-way analysis of variance, adjusted for sex and family type) (Hvolby et al. 2008). *ADHD* attention-deficit/hyperactivity disorder

A meta-analysis of four actigraphic studies reported statistically significantly longer mean sleep onset latency and shorter true sleep time in non-medicated children with ADHD than in typically developing controls (Cortese et al. 2009). Figure 1 illustrates data from one of these studies and shows that mean actigraphic sleep onset latency was longer in children with ADHD than in community controls and children with other psychiatric conditions (Hvolby et al. 2008). Mean longest sleep latency was also highest in children with ADHD, but total sleep time did not significantly differ among the three groups (Hvolby et al. 2008).

In subsequent actigraphic studies, differences between children with and without ADHD in sleep latency, sleep efficiency and total sleep time were statistically significant in one study (Moreau et al. 2013) but not in another (Wiebe et al. 2013). Waking after sleep onset was found to be increased in adolescents with ADHD (Mullin et al. 2011), and several actigraphic measures of sleep have also been reported to differ significantly in adults with ADHD, compared with controls (Boonstra et al. 2007; Gamble et al. 2013; Kooij et al. 2001; Van Veen et al. 2010). Actigraphic studies have also reported instability or increased night-to-night variability in sleep parameters in patients with ADHD compared with controls (Gruber and Sadeh 2004; Gruber et al. 2000; Hvolby et al. 2008; Moreau et al. 2013).

### Multiple sleep latency test

The MSLT provides a measure of daytime sleepiness by timing the first signs of sleep during daytime nap periods (Spruyt and Gozal 2011). The technique is subject to substantial heterogeneity, possibly due to differences in methodology and patient populations among studies (Cortese et al. 2009; Golan et al. 2004; Lecendreux et al. 2000). In meta-analyses (Cortese et al. 2006a, 2009), the average time taken to fall asleep in MSLTs was statistically significantly shorter in patients with ADHD than in controls, based on two included studies (Golan et al. 2004; Lecendreux et al. 2000). Both studies also reported that greater proportions of children with ADHD fell asleep during testing than did controls (Golan et al. 2004; Lecendreux et al. 2000). More recent studies have found no significant differences between children with ADHD and controls in MSLT outcomes (Prihodova et al. 2010; Wiebe et al. 2013), although one of these reported statistically significant inter-test variability in the ADHD group (Prihodova et al. 2010). There is also little agreement among three studies that have investigated the question of whether MSLT results correlate with objective measures of nocturnal sleep in individuals with ADHD (Golan et al. 2004; Lecendreux et al. 2000; Wiebe et al. 2013).

### Subjective assessments

Subjective measures of sleep in children are based on parent or child reports and include the BEARS sleep screening tool (Owens and Dalzell 2005), the Children’s Sleep Habits Questionnaire (CSHQ) (Owens et al. 2000), the Children’s Sleep Behavior Scale (CSBS) (Fisher et al. 1989) and sleep diaries (Hvolby et al. 2008, 2009). Adult instruments include the Pittsburgh Sleep Quality Index (Buysse et al. 1989). Instruments for subjective assessment of daytime sleepiness include the Epworth Sleepiness Scale.

The most recent meta-analysis of subjective studies found that ADHD was associated with statistically significant greater impairments in six parent-reported subjective measures of sleep in children than in controls (Cortese et al. 2009). The largest standardized mean difference was observed for bedtime resistance, followed by difficulty with morning awakenings, sleep onset difficulties, sleep-disordered breathing, night awakenings and daytime sleepiness. Other sleep problems reportedly associated with ADHD in children and/or adults include early and middle insomnia, nocturnal awakening, nocturnal activity, snoring, breathing difficulties, restless sleep, parasomnias, nightmares, daytime sleepiness, delayed sleep phase, short sleep time and anxiety around bedtime (Hansen et al. 2013; Hvolby et al. 2008, 2009; Spruyt and Gozal 2011; Yoon et al. 2012).

### Association of particular sleep problems with ADHD subtypes

Different patterns of sleep impairment may be characteristic of ADHD subtypes (Gruber 2009). Some studies show that parent-reported sleep disturbances are more common in combined-type ADHD than in predominantly inattentive ADHD (Corkum et al. 1999; Mayes et al. 2009), while others describe greater daytime sleepiness in predominantly inattentive ADHD than in combined-type ADHD (Chiang et al. 2010; LeBourgeois et al. 2004; Lecendreux et al. 2000). However, differences in symptom severity between subtypes may confound the associations with sleep problems (Corkum et al. 2011). Hyperkinetic disorder (HKD) diagnosed according to the ICD-10 criteria is generally regarded as a more severe form of combined-type ADHD than that described by the DSM, and children with HKD exhibited profound sleep-related problems in a study using subjective parent ratings (Gomes et al. 2013). If different sleep problems are associated with different ADHD subtypes, then this represents another potentially uncontrolled variable in studies investigating the relationship between sleep and ADHD (Kirov et al. 2004).

### Agreement and disagreement between subjective and objective measures of sleep

While laboratory studies are susceptible to artifacts (e.g., first-night effects), naturalistic studies are subject to uncontrolled variables such as parents’ work schedules, parenting style, family structure, child habits and employment in teenagers (Beebe 2011). Several studies have revealed discrepancies between results obtained using objective and subjective measures of sleep in patients with ADHD (Choi et al. 2010; Corkum et al. 2001; Hvolby et al. 2008; Lim et al. 2008; Owens et al. 2009; Wiggs et al. 2005; Yoon et al. 2012). In one study in children, parental estimates of sleep onset latency exceeded actigraphic estimates in about 75 % of cases, although mean sleep onset latency was longer in children with ADHD than in controls using both measures (Fig. 1) (Hvolby et al. 2008). Subjective reports may emphasize particularly problematic nights, which may not be captured in a single night’s objective measurement, or by averaging objective measurements over several nights: indeed, intra-individual variability in sleep parameters is reportedly higher in patients with ADHD than in controls (Gruber and Sadeh 2004; Gruber et al. 2000; Lecendreux and Cortese 2007; Moreau et al. 2013; Tsai and Huang 2010). Parental sensitivity to behavioral problems at bedtime may also lead to differences compared with objective assessments (Hvolby et al. 2008; Owens et al. 2009; Yoon et al. 2012). In adults with ADHD, self-reported sleep time, quality and efficiency were lower than in controls, but this was found to correlate with polysomnographic measures of PLMS and not with polysomnographic measures of sleep efficiency, length or onset latency (Philipsen et al. 2005). In summary, the available methods for assessing sleep each present their own advantages and disadvantages, with no single technique providing a complete picture of the complex interactions between sleep and ADHD.

## Relationship of sleep disorders to ADHD

Diagnosis of sleep disorders is based on formal subjective and/or objective criteria, such as the *International Classification of Sleep Disorders* (American Academy of Sleep Medicine 2005). Specific sleep disorders are associated with ADHD or ADHD-like symptoms, and systematic screening for sleep problems and disorders has been recommended during initial assessment and ongoing management of patients with ADHD (Cortese et al. 2013a). Inadequate sleep in children is known to have neurocognitive, neurobehavioral and functional manifestations that overlap with the core features of ADHD (O’Brien 2009; Owens et al. 2013). Experimental sleep restriction impacts on attention and higher-level cognitive function (Beebe 2011), and has been shown to affect neurobehavioral functioning in typically developing children (Gruber et al. 2011). No experimental study has yet shown that sleep restriction induces hyperactivity, impulsivity or externalizing behaviors in children (Beebe 2011), despite the perception that ‘paradoxical’ hyperactivity exists as a behavioral response to daytime sleepiness (Owens et al. 2013; Owens 2008). Recent observational studies in typically developing children have, however, shown that short sleep duration correlates with ADHD-like symptoms and behaviors scored by parents (Paavonen et al. 2009; Pesonen et al. 2010) and teachers (Gruber et al. 2012b).

### Sleep-disordered breathing and obstructive sleep apnea

The term sleep-disordered breathing (SDB) describes a spectrum of conditions ranging from obstructive sleep apnea (OSA) to primary snoring (O’Brien 2009; Owens 2008). SDB has been consistently associated with neurobehavioral and neurocognitive deficits, including inattentive or ADHD-like symptoms (Beebe 2006; Beebe et al. 2004; Chervin et al. 2002, 2012; Gottlieb et al. 2003; Lal et al. 2012; O’Brien 2009; Owens 2008; Rosen et al. 2004; Soylu et al. 2013; Suratt et al. 2011). Furthermore, a recent systematic review indicated that the prevalence of OSA in patients with ADHD (25–30 %) is higher than in the general population (about 3 %) (Youssef et al. 2011). Indeed, US guidelines recommend that children undergoing evaluation for ADHD are assessed for sleep apnea (Wolraich et al. 2011).

Surgical treatment of children with OSA via adenotonsillectomy in prospective, interventional studies has been reported to be associated with improvements in neuropsychological behavior (Beebe 2006), academic performance (Gozal 1998) and ADHD-like symptoms (Soylu et al. 2013; Youssef et al. 2011). In children with diagnoses of ADHD and OSA, two prospective studies have demonstrated significant improvements in ADHD symptoms, including hyperactivity, following adenotonsillectomy (Chervin et al. 2006; Huang et al. 2007). There is also some evidence that positive airway pressure ventilation in patients with OSA may also be associated with improvements in ADHD-like symptoms (Youssef et al. 2011). Large-scale, randomized, controlled studies are warranted to investigate further the effect of OSA treatment in patients with ADHD (Youssef et al. 2011).

### Restless legs syndrome and periodic limb movement disorder

Restless legs syndrome (RLS) is a neurological disorder characterized by an irresistible urge to move the legs to relieve uncomfortable sensations at rest (Picchietti and Picchietti 2010). Periodic limb movement disorder (PLMD) is a clinical syndrome characterized by PLMS of a specific nature and frequency determined by polysomnography (Picchietti and Picchietti 2010). While 2 % of typically developing children and adolescents (aged 8–17 years) are reported to meet the diagnostic criteria for RLS (Picchietti et al. 2007), up to 44 % of children with ADHD have symptoms of RLS, and 26 % of children with RLS have symptoms of ADHD (Cortese et al. 2005; Owens 2008). Accordingly, Cortese et al. have emphasized the importance of identifying RLS during clinical evaluation of children with ADHD symptoms (Cortese et al. 2006b). The recently revised diagnostic criteria for RLS in children introduced pediatric terms and prompts to allow the clinician to recognize typical descriptions of RLS symptoms, which must be in the child’s own words (Picchietti et al. 2013). As described previously, increased PLMS in patients with ADHD compared with controls is a common finding in polysomnographic studies. The impact of RLS or PLMD on sleep could lead not only to the diurnal manifestation of ADHD-like symptoms but also to bedtime resistance, which may be mistaken for opposition or defiance, due to the unpleasant symptoms (Cortese et al. 2006b).

### Circadian-rhythm sleep disorders

The major feature of circadian-rhythm sleep disorders is the misalignment of sleep pattern timing with the terrestrial cycle, leading to disrupted sleep and impaired functioning. In delayed sleep-phase disorder, sleeping and waking occur later than normal, and this may manifest as sleep onset insomnia, evening diurnal preference and difficulty waking. Such sleep problems are common, especially during adolescence: a meta-analysis of adolescent sleep studies revealed a worldwide delayed sleep–wake behavior pattern that was consistent with delayed sleep-phase disorder and resulted in decreased total sleep time and daytime sleepiness (Gradisar et al. 2011). There is evidence to suggest that ADHD may be associated with disturbances of the circadian rhythm. A delayed pattern of melatonin secretion in children with ADHD compared with controls has been described (Van der Heijden et al. 2005, 2007). Children with ADHD have also been reported to exhibit stronger circadian evening tendencies than controls, as assessed using the child morning-evening preference scale. Scores on this parent-rated instrument were correlated with both parental and polysomnographic measures of sleep onset latency (Gruber et al. 2012a). In adults with ADHD, disturbances in diurnal rhythms of endocrine secretion, *CLOCK* gene expression and physical activity have been reported (Baird et al. 2012; Bijlenga et al. 2013). Furthermore, delayed sleep timing in adults with ADHD and comorbid insomnia compared with controls has been documented (Van Veen et al. 2010) and shown to correlate with the severity of ADHD symptoms (Gamble et al. 2013).

### Interaction of obesity with sleep disorders and ADHD

There is good evidence from cross-sectional studies for an association of ADHD with obesity: The prevalence of ADHD is higher than expected in people with obesity sampled at obesity clinics, and the body mass index of patients with ADHD is higher than average (Cortese et al. 2008a). Furthermore, in a 33-year longitudinal study, 41.4 % of adults who had combined-type ADHD as children were obese, compared with 21.6 % of those without a childhood diagnosis of ADHD (Cortese et al. 2013c). Obesity is, in turn, correlated with sleep-disordered breathing and other sleep disorders (Cortese et al. 2008b), short sleep duration (Taheri et al. 2004) and short time in bed (Hart et al. 2013). Abnormal eating behaviors associated with ADHD (e.g., impulsive eating) might contribute to obesity (Cortese and Vincenzi 2012), and in adolescents with obesity but without diagnosed ADHD, daytime sleepiness has been reported to correlate with ADHD symptom ratings (Cortese et al. 2007). Together, these data suggest a complex interplay between ADHD, obesity and sleep problems.

## Effects of ADHD medications on sleep

ADHD medications are known to affect sleep in many individuals, and guidelines recommend that sleep is carefully assessed before starting ADHD pharmacotherapy (Graham et al. 2011; Wolraich et al. 2011). Sleep disturbances in patients with ADHD, including those associated with ADHD medications, may be addressed via pharmacological and behavioral interventions, with the latter forming part of the recommended multimodal strategy (Cortese et al. 2013a).

### Pharmacotherapy with stimulants

The effects of stimulants on sleep in patients with ADHD differ from patient to patient and reflect the underlying complexity of the links between ADHD and sleep disturbance (Graham et al. 2011). The sympathomimetic action of stimulants promotes wakefulness in most people, underlying their use in the treatment of narcolepsy (Morgenthaler et al. 2007). While there is evidence that stimulants are associated with disrupted or disturbed sleep in patients with ADHD (Ironside et al. 2010; Nutt et al. 2007; Spruyt and Gozal 2011; Stein 1999), clinical experience also indicates that stimulants produce paradoxical effects (Bradley 1937), whereby alleviation of symptoms can calm patients and promote sleep (Jerome 2001; Kinsbourne 1973; Kooij et al. 2001; Kratochvil et al. 2005). Furthermore, because of the potential for symptom rebound as blood drug concentrations wane (Carlson and Kelly 2003), an additional dose of a short-acting stimulant, or the use of a formulation with an increased duration of action, may prevent sleep disturbances resulting from worsening of hyperactivity or behavioral difficulties at bedtime (Cortese et al. 2013a, b; Lecendreux et al. 2000).

In clinical trials using objective sleep measures, immediate-release methylphenidate has been reported to increase sleep onset latency and/or to decrease total sleep time in patients with ADHD (Boonstra et al. 2007; Galland et al. 2010; Greenhill et al. 1983; Sangal et al. 2006), with sleep quality either unaffected (Galland et al. 2010) or improved (Boonstra et al. 2007; Sobanski et al. 2008). A recent meta-analysis of six actigraphic studies in children with ADHD reported statistically significant lower daytime activity, longer sleep onset latency, lower total sleep time and lower sleep efficiency with immediate-release methylphenidate treatment than with placebo (De Crescenzo et al. 2014). Both amfetamine and methylphenidate are associated with treatment-emergent adverse events (TEAEs) of insomnia in clinical studies (Efron et al. 1997; Stein et al. 2011).

Long-acting stimulants are available in many different formulations (Hodgkins et al. 2012). This section focuses on osmotic-release oral system methylphenidate (OROS-MPH) and lisdexamfetamine dimesylate (LDX), both of which are recently developed and widely used ADHD medications with daily durations of efficacy of at least 12 h post-dose (Coghill and Seth 2006; Lakhan and Kirchgessner 2012; Setyawan et al. 2013a, b; Steer et al. 2012).

OROS-MPH combines an immediate-release bolus with a two-stage extended-release technology to provide an ascending profile of drug delivery similar to three daily doses of immediate-release methylphenidate (Swanson et al. 2003). In an open-label polysomnographic study in children with ADHD, the only statistically significant effects of OROS-MPH treatment were a decrease in the number of night-time awakenings and an increase in the percentage of stage 2 sleep, compared with pre-treatment baseline (Kim et al. 2010). Based on parental sleep diaries, both OROS-MPH and another extended-release methylphenidate formulation led to statistically significant reductions in total sleep time 1–4 weeks after initiation of treatment in a randomized study in children with ADHD (Lee et al. 2012). In a randomized, double-blind, placebo-controlled study, the majority of children in all three treatment groups (OROS-MPH, placebo or immediate-release methylphenidate three times daily) continued to have good or excellent sleep quality based on parent ratings at 2 and 4 weeks after initiation of treatment (Wolraich et al. 2001). Sleep quality was also rated as good or excellent by parents after 1 and 12 months of open-label OROS-MPH treatment in children with ADHD (Wilens et al. 2003). Table 1 shows a summary of the proportions of patients experiencing TEAEs of insomnia in randomized, double-blind, placebo-controlled, parallel-group clinical studies of OROS-MPH.

**Table 1:** Frequency of TEAEs of insomnia (or similar) in randomized, double-blind, placebo-controlled, parallel-group clinical studies of OROS-MPH in patients with ADHD

Study	Age of population, years	Duration, weeks	Treatment (*n*)	Proportion of patients reporting a TEAE, %
Medori et al. (2008)	18–65	5	Placebo (96)	7.3
			OROS-MPH (305)	13.4
Biederman et al. (2006)	19–60	6	Placebo (74)	5^a^
			OROS-MPH (67)	18^a^
Biederman et al. (2010)	19–60	6^b^	Placebo (109)	4^c^
			OROS-MPH (114)	11^c^
Adler et al. (2009b)	18–65	7	Placebo (116)	5.2 (3.4)^d^
			OROS-MPH (110)	9.1 (7.3)^d^
Newcorn et al. (2008)	6–16	6	Placebo (74)	1^e^
			OROS-MPH (219)	13^e^
			Atomoxetine (221)	7^e^
Findling et al. (2008)	6–12	7	Placebo (85)	4.7
			OROS-MPH (91)	7.7
			Transdermal methylphenidate (98)	13.3
Casas et al. (2013)	18–65	13	Placebo (97)	11.3 (2.1)^d^
			OROS-MPH 54 mg (89)	14.6 (7.9)^d^
			OROS-MPH 72 mg (92)	16.3 (9.8)^d^

Randomized-withdrawal studies are excluded

*ADHD* attention-deficit/hyperactivity disorder, *OROS*-*MPH* osmotic-release oral system methylphenidate, *TEAE* treatment-emergent adverse event

^a^Frequency of ‘sleep problems’

^b^Acute efficacy phase

^c^TEAEs reported on two or more visits

^d^Frequency of initial insomnia

^e^Includes insomnia, initial insomnia, middle insomnia and late insomnia

**Fig. 2:**
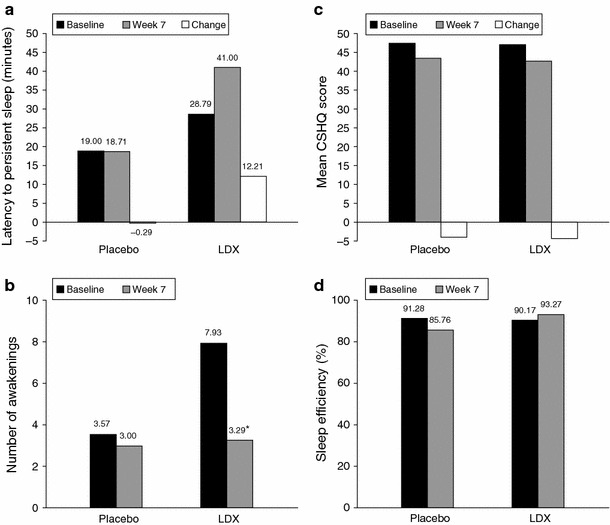
**a**, **b** Polysomnographic, **c** parent-rated subjective and **d** actigraphic outcomes from a double-blind, randomized, parallel-group study of the effects of LDX treatment on sleep in 24 children with ADHD (Giblin and Strobel 2011). *p < 0.0001 versus baseline. *ADHD* attention-deficit/hyperactivity disorder, *CSHQ* Children’s Sleep Habits Questionnaire, *LDX* lisdexamfetamine

LDX is the only stimulant prodrug. After oral administration, rate-limiting enzymatic hydrolysis of LDX in the bloodstream releases the pharmacologically active *d*-amfetamine moiety from the lysine conjugate (Steer et al. 2012). LDX treatment was not associated with impairments in sleep quality or quantity in clinical trials using objective sleep measures in adults with ADHD (Adler et al. 2009a; Surman and Roth 2011) and children with ADHD (Giblin and Strobel 2011) (Fig. 2). Table 2 shows a summary of the proportions of patients experiencing TEAEs of insomnia in randomized, double-blind, placebo-controlled, parallel-group clinical studies of LDX.

**Table 2:** Frequency of TEAEs of insomnia (or similar) in randomized, double-blind, placebo-controlled, parallel-group clinical studies of LDX in patients with ADHD

Study	Age of population, years	Duration, weeks	Treatment (*n*)	Proportion of patients reporting a TEAE, %
Biederman et al. (2007)	6–12	4	Placebo (72)	2.8
			LDX (218)	18.8
Adler et al. (2008)	18–55	4	Placebo (62)	5
			LDX (358)	17–21^a^
Findling et al. (2011)	13–17	4	Placebo (77)	3.9
			LDX (223)	11.2
Coghill et al. (2013)	6–17	7	Placebo (110)	0.0 (0.9)^c^
			LDX (111)	14.4 (2.7)^c^
			OROS-MPH (111)^b^	8.1 (6.3)^c^
Adler et al. (2013)	18–55^d^	10	Placebo (80)	3.8
			LDX (79)	12.7

Randomized-withdrawal studies are excluded

*ADHD* attention-deficit/hyperactivity disorder, *LDX* lisdexamfetamine dimesylate, *TEAE* treatment-emergent adverse event

^a^Range across forced-dose groups (30, 50 or 70 mg/day)

^b^Reference arm (active control)

^c^Frequency of initial insomnia

^d^Patients with ADHD and executive function deficits

### Pharmacotherapy with non-stimulants

In contrast to stimulants, somnolence is the most common sleep-related adverse event associated with atomoxetine (a noradrenaline reuptake inhibitor approved for treatment of ADHD). In a 2009 systematic review, the frequency of somnolence reported as a TEAE in placebo-controlled clinical trials of atomoxetine was reported to range from 15 to 17 % (Garnock-Jones and Keating 2009). In a randomized, double-blind trial, atomoxetine was associated with a smaller increase in sleep onset latency, a lower frequency of insomnia, a higher frequency of somnolence and smaller effects on subjective measures of sleep than methylphenidate taken three times daily (Sangal et al. 2006). Lower frequencies of insomnia and higher frequencies of somnolence with atomoxetine than with long-acting stimulants have been reported as TEAEs in randomized, double-blind, parallel-group efficacy studies (Dittmann et al. 2013; Newcorn et al. 2008). Dosing in the evening rather than in the morning has been found to reduce daytime somnolence with atomoxetine (Block et al. 2009).

An extended-release formulation of guanfacine, a selective α_2_-adrenoceptor agonist, is approved in North America for the treatment of children and adolescents with ADHD, both as a monotherapy and as an adjunct to stimulant treatment. Somnolence is one of the most commonly reported TEAEs in clinical trials of extended-release guanfacine (either alone or when co-administered with a stimulant) (Faraone et al. 2013). The effects of guanfacine on ADHD symptoms have been suggested to be independent of its sedative properties (Kollins et al. 2011). Extended-release clonidine (another selective α_2_-adrenoceptor agonist) is also approved in North America with a similar indication (Childress and Sallee 2012) and is associated with somnolence (Cortese et al. 2013b).

## Management of sleep problems in patients with ADHD

Both European and US guidelines recommend assessment of sleep disturbance during evaluation of an individual for suspected ADHD, and before initiation of pharmacotherapy (Graham et al. 2011; Wolraich et al. 2011). This approach enables any effects of the disorder on sleep to be distinguished from those of medication (Cortese et al. 2013b). Clinicians have been advised to use sleep diaries and questionnaires for routine screening and follow-up, together with specific screening for RLS and polysomnography when a physical sleep disorder is suspected (Cortese et al. 2013b). In developing a multimodal treatment plan for patients with ADHD, consideration should be given to interventions focused on improving sleep and bedtime behavior (Lecendreux and Cortese 2007). Both non-pharmacological and pharmacological interventions are available for improving sleep in patients with ADHD, and are applicable to sleep disturbance associated with ADHD medication and with the disorder itself. Potential strategies for managing sleep disturbances during treatment with ADHD medications are summarized in Table 3.

**Table 3:** Recommended strategies for managing sleep disturbances during treatment with ADHD medications (Cortese et al. 2013b)

Monitoring: insomnia associated with stimulants may attenuate after 1–2 months (Lecendreux and Cortese 2007)
Considering if it is possible to stop the medication
Implementing sleep hygiene/behavioral measures
Reviewing the possible causes of sleep problems
Treating RLS
Adding small, short-acting stimulant doses in the early evening (if rebound effect occurs)
Reducing stimulant dose
Switching to an alternative class of stimulant
Switching to an alternative stimulant formulation
Considering use of a non-stimulant (e.g., atomoxetine)
Considering melatonin treatment

*ADHD* attention-deficit/hyperactivity disorder, *RLS* restless legs syndrome

### Sleep hygiene

Healthy sleep practices include the following: a regular sleep/wake schedule; adequate opportunity for sleep; calming and structured bedtime routines; avoidance of caffeine, large amounts of liquids, naps, exercise and alerting activities (e.g., use of electronic devices) soon before bedtime; sleeping only in bed and using the bed only for sleeping; and attention to environmental factors such as bedroom furniture, lighting and temperature (Cortese et al. 2013a; Owens 2008; Yoon et al. 2012). In a study of children with ADHD and initial insomnia receiving stimulants, implementing sleep hygiene reduced sleep onset delay to below 60 min in about 20 % of patients, with an overall effect size of 0.67 (Weiss et al. 2006). Implementing healthy sleep practices is the recommended first-line option for addressing problems with sleep in both medicated and un-medicated patients with ADHD (Cortese et al. 2013b; Lecendreux and Cortese 2007).

### Behavioral interventions

Established behavioral interventions for insomnia in typically developing children include parent education, graduated extinction (ignoring disruptive behaviors for a predetermined period) and bedtime fading, which involves identifying a bedtime at which the child falls asleep within about 15 min, and gradually setting bedtime earlier until the desired bedtime is achieved, while keeping wake time fixed and disallowing sleep at other times (Mindell et al. 2006; Vriend and Corkum 2011). Clinical studies of behavioral interventions to improve sleep in children with ADHD are limited and have not demonstrated any effect on ADHD symptoms (Cortese et al. 2013a). A pilot study indicated that a sleep program involving face-to-face and telephone contact with a specialist pediatrician or child psychiatrist improved children’s sleep, quality of life and psychosocial functioning, based on parent reports after 5 months (Sciberras et al. 2011). This approach is under evaluation in a larger randomized, controlled study (Sciberras et al. 2010). Case reports also indicate efficacy of behavioral programs in reducing the severity of dyssomnia in children with ADHD (Mindell et al. 2006).

### The ball blanket

**Fig. 3:**
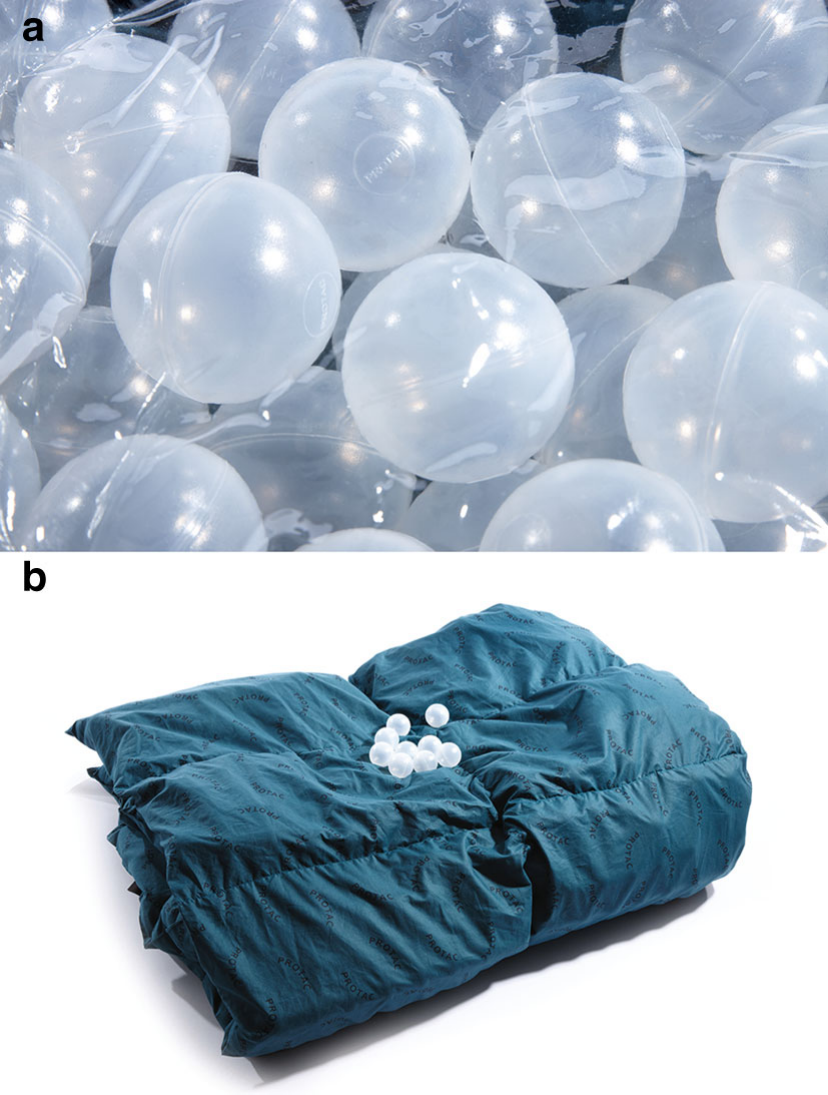
Ball blanket. **a** Plastic balls, diameter 49 mm and **b** cotton blanket containing 7 kg of balls and measuring 140 × 200 cm

Ball blankets (Fig. 3) are filled with loose balls to stimulate sensory receptors in the skin, muscles and joints, which transmit inhibitory signals to the central nervous system (Hvolby and Bilenberg 2011). In a study in children with ADHD, the use of ball blankets was found to reduce sleep onset latency, the number of awakenings and intra-individual variability in sleep parameters (Hvolby and Bilenberg 2011).

### Pharmacological strategies

In addition to adjusting dose, class, formulation or regimen of ADHD medications, sleep problems in patients with ADHD may be addressed via additional medications (Table 3). In patients with psychiatric comorbidities, it should be borne in mind that other medications (e.g., antidepressants) may also affect sleep. Implementation of healthy sleep practices should precede pharmacological interventions targeted at specific sleep disorders in patients with ADHD (Cortese et al. 2013a).

Iron deficiency has been implicated in the etiology of both RLS and ADHD, with potential links to alteration of dopamine transporter expression and the synthesis and catabolism of monoaminergic neurotransmitters (Allen and Earley 2007; Cortese et al. 2005, 2012). A small, randomized study of iron supplementation in children with ADHD detected a statistically significant reduction of ADHD symptoms (Konofal et al. 2008). Monitoring serum ferritin levels has been proposed for children with suspected RLS (Picchietti and Picchietti 2010), and there is some evidence that iron supplementation may be effective in relieving the symptoms of RLS in children (Cortese et al. 2013a). The role of monoamine neurotransmission in ADHD and RLS was investigated more directly in a double-blind, placebo-controlled trial in 29 children with ADHD or ADHD and RLS/PLMS. In this study, levodopa treatment slightly improved PLMS and/or RLS symptoms, but did not affect other sleep parameters, ADHD symptoms or performance in neuropsychometric tests (England et al. 2011). However, a subsequent subgroup analysis of this study failed to confirm the effect of levodopa on PLMS (Ferri et al. 2013). These results suggest that further work is needed to unravel the relationship, if any, between dopamine, ADHD and RLS/PLMS. No therapies have yet received regulatory approval for treating RLS in children (Cortese et al. 2013a).

Patients with ADHD and circadian-rhythm disorder are reported to exhibit a delayed pattern of melatonin secretion. Melatonin is classified as a dietary supplement in the USA but is subject to drug regulation in Europe (Bendz and Scates 2010). Two randomized, double-blind, placebo-controlled trials (Van der Heijden et al. 2007; Weiss et al. 2006) and a preliminary open-label study (Tjon Pian Gi et al. 2003) have indicated that melatonin treatment is effective in reducing sleep onset delay in children with ADHD (Cortese et al. 2013a).

Hypnotic agents, including zolpidem, mirtazapine, trazodone and antihistamines, have been used off-label in clinical practice to treat insomnia in children with ADHD (Kratochvil et al. 2005) and some have been evaluated in clinical trials (Cortese et al. 2013b), but their use does not form part of current clinical guidelines. Clonidine has also been suggested as a treatment option for stimulant-associated sleep onset delay in patients with ADHD (in an immediate-release formulation rather than the extended-release formulation used as an ADHD therapy) (Prince et al. 1996; Wilens et al. 1994).

Real-world data on sleep medication use in patients with ADHD is scarce. In observational study in a population of children with ADHD, 63 % of whom had moderate or severe sleep problems, 19 % took clonidine and 9 % took melatonin during the 1-week reporting period (none took antihistamines, benzodiazapenes or dopamine agonists) (Efron et al. 2014).

## A conceptual model of the interactions of ADHD with sleep

**Fig. 4:**
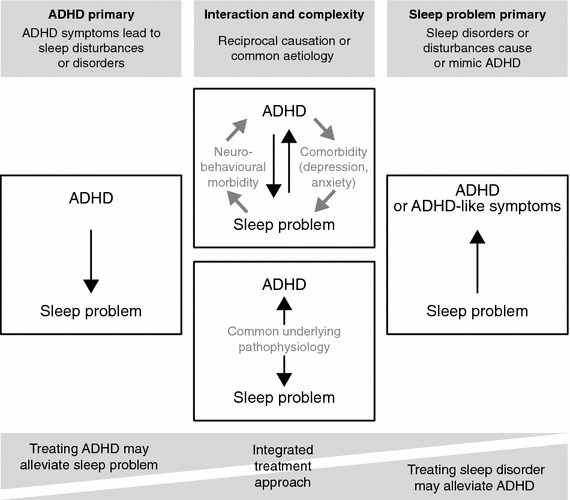
Conceptual model of the modes of interaction between ADHD and sleep. *ADHD* attention-deficit/hyperactivity disorder

Associations between ADHD and sleep disorders and subjective or objective measures of sleep or sleep disturbance do not provide information on causation. As an interpretative aid, this section presents a conceptual model of the potential relationships between sleep problems and ADHD or ADHD-like symptoms (Fig. 4). This theoretical framework is made up of four hypothetical scenarios.

In one scenario, ADHD leads directly to problems with sleep (Fig. 4, left-hand panel). This may be due to hyperactivity, nocturnal motricity or behavior (e.g., bedtime resistance). This scenario may be more pertinent to patients with hyperactive symptoms than to those with predominantly inattentive ADHD. If sleep problems are a consequence of ADHD symptoms, treatment with stimulants may help a patient to sleep by reducing these symptoms. Insufficient duration of efficacy may, however, lead to symptom rebound at bedtime.

In a contrasting scenario, disturbed sleep is responsible for daytime symptoms, behaviors and functional impairments that are characteristic of ADHD (Fig. 4, right-hand panel). The strongest evidence for a sleep disorder giving rise to ADHD or ADHD-like symptoms is the amelioration of such symptoms after surgical intervention to improve nocturnal breathing. It has been recommended that primary sleep disorders are excluded before diagnosing ADHD (Cortese et al. 2006b, 2013a; Lecendreux and Cortese 2007). In this situation, psychostimulant medications might be ineffective or could even exacerbate sleep problems. In contrast, treating an underlying sleep disorder could result in daytime improvements (O’Brien 2009). That successful treatment of OSA, RLS and delayed sleep-phase disorder can lead to improvements in ADHD symptoms is borne out by case studies (Miano et al. 2013).

In another scenario, sleep disturbances and ADHD are coincident, but may exacerbate each other in a feed-forward loop (Fig. 4, upper-middle panel). Individuals with ADHD could be both more vulnerable to the effects of sleep disturbance and more prone to disturbed sleep than typically developing children (Owens et al. 2013). The choice of treatment in this situation is complex because medications could have opposing or mixed effects on sleep. Psychiatric comorbidities are common in children with ADHD, and these may be associated with sleep problems. Furthermore, daytime sleepiness has been reported to be associated with worsened internalizing symptoms in children with anxiety disorder, suggesting that poor sleep may negatively affect emotional regulation as well as attentional functioning (Hansen et al. 2013). The possible interplay between sleep, ADHD and anxiety in children may be related to the alterations observed in sleep-deprived individuals in overlapping brain mechanisms involved in alertness and reward pathways (Gruber 2014). Sleep disturbances in ADHD can, however, occur independently of psychiatric comorbidities, as demonstrated in studies employing psychiatric control groups (Hvolby et al. 2008). Nevertheless, comorbid psychiatric disorders (both internalizing and externalizing) may further exacerbate both sleep problems and ADHD symptoms in patients with ADHD.

In a final scenario, common or overlapping neurobiological disease mechanisms are hypothesized to give rise to both ADHD and sleep disturbance (Fig. 4, lower-middle panel). Circadian-rhythm disorders, sleep/wake disorders and delayed sleep-phase disorder could share pathophysiological mechanisms with ADHD. There may be a genetically determined predisposition to sleep dysregulation in at least a subset of individuals with ADHD (Owens et al. 2013). Intra-individual variability in neuropsychological tasks, rather than a constant level of impairment, is characteristic of ADHD (Spencer et al. 2009; Tamm et al. 2012), and a similar picture of volatility and unpredictability is observed in sleep patterns in children with ADHD (Gruber and Sadeh 2004; Gruber et al. 2000; Lecendreux and Cortese 2007; Tsai and Huang 2010). Furthermore, sleep duration decreases during development, and a more rapid decrease compared with normative centiles at 3–5 years of age has been reported to be a significant predictor of subsequent ADHD (Scott et al. 2013).

## Conclusions

ADHD is commonly associated with specific sleep disorders and objectively or subjectively assessed sleep disturbances. The relationship between ADHD and sleep problems is complex and bidirectional, and is modulated by interactions with ADHD medications and by psychiatric comorbidities and associated medications. Understanding these associations and relationships is important when assessing and managing patients with ADHD. As recommended in current guidelines, primary sleep disorders (specifically SDB/OSA and PLMD/RLS) should be ruled out before diagnosing or treating ADHD. Obesity and psychiatric comorbidities (e.g., anxiety and depression) can also lead to sleep problems, and need to be identified and treated appropriately. The multifaceted effects of stimulant pharmacotherapy on sleep in patients with ADHD are particularly important for clinicians to understand when evaluating treatment options for patients. Stimulant medications may disrupt or improve sleep in different patients, depending not only on the nature of the patient’s illness, but also on the drug dose, class, formulation and duration of efficacy. Effective management of sleep problems associated with ADHD and its treatment may not only alleviate sleep-related symptoms, but also improve quality of life in parents or carers of children with disruptive bedtime behavior or insomnia.

In the near term, new understanding of how sleep interacts with ADHD and how this affects treatment choices is likely to come from clinical studies. Polysomnographic studies need to be larger and better controlled than previous studies, and should not overlook subtle polysomnographic signals such as microarousals and the time structure of PLMS. There is also a need to follow up the recent data indicating a link between ADHD, obesity, daytime sleepiness and circadian-rhythm alterations. Conspicuously absent from the current literature is any convincing demonstration that pharmacological or non-pharmacological intervention to improve sleep actually leads to improved ADHD symptoms or reduced functional impairment associated with the disorder. Similarly, as children become adolescents, they may experience sleep loss, but do any adolescents benefit from more or better sleep in terms of preventing worsening of ADHD? Crucial to both these questions is the possibility that specific ADHD phenotypes are associated with, or characterized by, particular types of sleep-related problems. In addition to helping clinicians make treatment decisions, the ability to subcategorize patients with ADHD based on their sleep phenotype may help shed light on areas where current data are conflicting. Furthermore, such phenotypic classification is probably essential for increased sensitivity in genomic screens for ADHD-associated polymorphisms. The overlapping neurochemical and neuroanatomical systems involved in regulating sleep, attention, arousal and circadian rhythms are the subjects of current basic research (Owens et al. 2013). Whether enhanced understanding of these mechanisms in health and disease or the use of state-of-the-art genetic and neuroimaging tools will lead to the development of new therapies or preventative strategies for ADHD are questions for the future.
